# Potential Use of Exosomal Non-Coding MicroRNAs in Leukemia Therapy: A Systematic Review

**DOI:** 10.3390/cancers16233948

**Published:** 2024-11-25

**Authors:** Paulina Gil-Kulik, Natalia Kluz, Dominika Przywara, Alicja Petniak, Małgorzata Wasilewska, Natalia Frączek-Chudzik, Marek Cieśla

**Affiliations:** 1 Department of Clinical Genetics, Medical University of Lublin, 11 Radziwillowska Str., 20-080 Lublin, Poland; nataliakluz99@gmail.com (N.K.); dprzywara17@gmail.com (D.P.); alicja.petniak@umlub.pl (A.P.); 2 Department of Gastroenterology and Internal Medicine, Medical University of Warsaw, 02-091 Warsaw, Poland; 3 Department of Physical Chemistry, Institute of Chemical Sciences, Faculty of Chemistry, Maria Curie-Sklodowska University in Lublin, Maria Curie-Sklodowska Sq. 3, 20-031 Lublin, Poland; malgorzata.wasilewska@mail.umcs.pl; 4 Institute of Medical Science, College of Medical Science, University of Rzeszow, 35-959 Rzeszow, Poland; natalia_from@wp.pl (N.F.-C.); mciesla@ur.edu.pl (M.C.)

**Keywords:** ncRNA, microRNA, leukemia, therapy, exosomes

## Abstract

Leukemia is a heterogeneous group of hematological malignancies. Despite the enormous progress that has been made in the field of hemato-oncology in recent years, there are still many problems related to, among others, disease recurrence and drug resistance, which is why the search for ideal biomarkers with high clinical utility continues. Research shows that exosomes play a critical role in the biology of leukemia and are associated with the drug resistance, metastasis, and immune status of leukemias. Exosomes with their cargo of non-coding RNAs act as a kind of intermediary in intercellular communication and, at the same time, have the ability to manipulate the cell microenvironment and influence the reaction, proliferative, angiogenic, and migratory properties of cells. Exosomal ncRNAs (in particular, circRNAs and microRNAs) appear to be promising cell-free biomarkers for diagnostic, prognostic, and treatment monitoring of leukemias. This review examines the expression of exosomal ncRNAs in leukemias and their potential regulatory role in leukemia therapy but also in conditions such as disease relapse, drug resistance, metastasis, and immune status. Given the key role of ncRNAs in regulating gene networks and intracellular pathways through their ability to interact with DNA, transcripts, and proteins and identifying their specific target genes, defining potential functions and therapeutic strategies will provide valuable information.

## Simple Summary

This review shows the expression of exosomal microRNAs in leukemias and their potential regulatory role in leukemia therapy. The main objective is to evaluate the potential use of exosomal-derived non-coding RNAs from the point of view of clinical application as therapeutic agents in the treatment of leukemia. Exosomal miRNAs such as miR-21, miR-23b-5p, miR-34, miR101-3p, miR-150, and miR-155 appear to have the greatest therapeutic potential.

## 1. Introduction

Leukemia is a prevalent malignant disease. It is estimated that over 487,000 individuals worldwide were diagnosed with leukemia in 2022, resulting in approximately 305,000 deaths [1,2,3]. Furthermore, it is the most frequently diagnosed cancer in children under the age of five, accounting for 30% to 40% of all pediatric tumors [4]. It affects the bone marrow, lymphatic system, blood-forming organs, and spleen [5]. According to the Fourth Edition of the WHO Classification of Tumors of Hematopoietic and Lymphoid Tissues, leukemia can be broadly classified into myeloid or lymphoid lineages [6]. There are four predominant subtypes of leukemia—acute lymphoblastic leukemia (ALL), acute myeloid leukemia (AML), chronic lymphocytic leukemia (CLL), and chronic myeloid leukemia (CML) [7]. Of the acute leukemias, ALL is commonly diagnosed in children and young adults, with a peak incidence at 2 to 5 years of age [8], while AML is the most common acute form in adults, accounting for 1.3% of new cancer cases in the USA [9]. Over the past few decades, progress in the treatment of hematopoietic malignancies has been particularly rapid due to improvements in treatment protocols, including the development of targeted therapies such as tyrosine kinase inhibitors (TKIs) [10,11]. However, despite huge improvements in the treatment of leukemia in recent years, some patients develop intrinsic or acquired resistance to treatment during the course of their disease [12]. In leukemia and other hematologic malignancies, extracellular vesicles (EVs) have been shown to participate in the growth of the primary tumor and also to induce resistance to many drugs by recruiting resident cells of the microenvironment, such as endothelial cells (ECs) or leukocytes [13].

EVs constitute a heterogeneous group of membrane-bound particles released by cells into the extracellular environment. The three most widely recognized subtypes are typically designated as exosomes (30–150 nm in diameter), microparticles (also known as microbubbles, 100–1000 nm in diameter), and apoptotic bodies (50 to 5000 nm in diameter) [14,15]. They are released into the extracellular environment by a variety of cell types and serve a pivotal role in intercellular communication. Exosomes transport a diverse array of bioactive molecules, including proteins, lipids, and nucleic acids (DNA, RNA, and non-coding RNA) from the cell of origin to recipient cells, thereby influencing a multitude of physiological and pathological processes [14]. Exosomes have been observed to be present in greater quantities in the blood of cancer patients than in that of healthy individuals, which suggests a potential correlation between cancer and increased exosome secretion [16]. These exosomes, secreted by a tumor, may interact with neighboring cells or the extracellular matrix or may be transported passively through the bloodstream and other body fluids [17].

**Figure 1:**
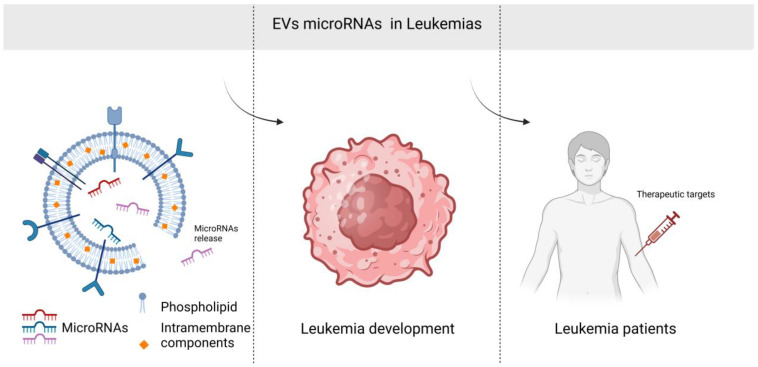
EV–miRNAs in leukemias (created in BioRender.com (accessed on 3 November 2024), license number SQ27I5OIMZ), https://BioRender.com/r51c603 accessed on 3 November 2024.

Exosomal non-coding RNAs (ncRNAs), including microRNAs (miRNAs, miRs), long non-coding RNAs (lncRNAs), and circular RNAs (circRNAs), have significant therapeutic potential in leukemia due to their ability to regulate gene expression, intercellular communication, and involvement in key oncogenic pathways. As natural carriers of ncRNAs, exosomes provide a distinctive platform for the development of innovative therapeutic strategies for leukemia [18]. Moreover, exosomal ncRNAs display distinct expression profiles in different cells or in different physiological and pathological states, indicating the potential involvement of these exosomal biomolecules in the pathogenesis and progression of a wide range of diseases. Consequently, ncRNAs are emerging as promising diagnostic and therapeutic tools for a plethora of human diseases, including leukemia [19]. Among the RNAs encapsulated in EVs derived from human plasma, miRNAs are the most abundant [20]. miRNAs are a class of small ncRNAs comprising approximately 22 nucleotides. They play a pivotal role in regulating gene expression. In the context of leukemia, specific miRNAs can function as oncogenes (oncomiRs) or tumor suppressors. Exosomes are capable of delivering miRNAs to recipient cells, thereby influencing the leukemia microenvironment or modifying the behavior of cancer cells [17] (Figure 1). Overexpressed miRNAs in leukemia such as miR-21, miR-155, and miR-19a/b affect tumorigenesis and the proliferation of cancer cells. These miRNAs prevent apoptosis and support the survival and growth of leukemic cells. In addition, miR-21, miR-125b, and miR-155 are directly involved in drug resistance. The overexpression of miRNAs such as miR-223 and miR-146a modifies the tumor microenvironment [21,22,23]. The downregulation of specific miRs in leukemia, including miR-34a, miR-29b, and miR-145, has been demonstrated to influence apoptosis and cell survival. Furthermore, the downregulation of specific miRNAs in EVs, including the miR-142a and miR-34a, has been demonstrated to promote the stemness of leukemic cells, thereby contributing to the persistence of leukemic stem cells, which are often resistant to therapy and responsible for relapse [24,25]. Decreased miRNAs, such as miR-150 and miR-181a, have been shown to modulate the immune response by targeting genes that regulate T-cell activity and immune surveillance [21,23].

The aim of this systematic review was to highlight the regulatory role of individual EV-derived miRNA molecules in leukemia therapy and to summarize the existing knowledge of their mechanisms of action. The focus was on the potential use of exosomal-derived ncRNAs, especially miRNA, from the point of view of clinical application as therapeutic agents in the treatment of leukemia and on the properties that contribute to various pathological processes in leukemia, including disease progression, drug resistance, metastasis, relapse, and immune modulation.

## 2. Methods

### 2.1. Protocol

Although a formal review protocol was not pre-registered for this study, the methodological framework was guided in accordance with the Preferred Reporting Items for Systematic Reviews and Meta-analyses (PRISMA) guidelines https://www.prisma-statement.org/prisma-2020-statement [26] accessed on 30 September 2024.

This systematic review was performed in line with PRISMA guidelines. All items included in the PRISMA checklist are covered in the respective subsections of the article.

This systematic review has been registered at: https://osf.io/rfs7t/ DOI:10.17605/OSF.IO/RFS7T (accessed on 30 September 2024)

### 2.2. Data Sources and Search Strategy

A comprehensive electronic search was performed by the reviewer across multiple databases to identify relevant evidence on the topic: PubMed (Medline), Cochrane, and Scopus. The PICO framework focused on population, intervention, comparison, and outcomes. 

The population included patients suffering from various types of leukemia, as well as animal models and cell lines. The intervention involved examining the status of exosomal microRNA and its functions in these malignancies. The comparison considered the relationship between clinicopathological characteristics and microRNA expression. The outcome was centered on the diagnostic utility and molecular function in hematological malignancies. 

The search excluded unpublished studies and gray literature, emphasizing high-quality, peer-reviewed evidence. The strategy utilized a combination of MeSH terms and Boolean operators, with various formats and synonyms related to the key concepts of microRNAs from exosomes and hematological malignancies. The strategy was slightly adapted for each database to ensure the broadest but still relevant range of results. Publications from 2014 to 2024 were included, and the main keywords used were microRNA, miRNA, leukemia, exosomes, and RNA, untranslated. Each concept was represented by different synonyms and abbreviations, with Boolean operators (OR and AND) used to ensure comprehensive retrieval from the selected databases. 

The search strategy was customized to the specific features of each database to optimize results. For example, the PubMed search string was as follows: (microrna OR mirna OR RNA, untranslated) AND (exosomes) AND (leukemia). The last publication was found on 7 September 2024.

### 2.3. Eligibility Criteria

Given its nature as a scoping review, this study applied broad eligibility criteria to capture the maximum number of relevant studies on the role of microRNAs in various types of leukemia with a particular focus on the potential use of exosomal microRNA in leukemia therapy.

The review encompassed studies related to the detection, characterization, quantification, and functional analysis of microRNAs in these disorders, aiming to identify specific microRNA types, quantities, and functions. Non-English language publications were excluded to ensure accuracy and to minimize potential translation errors. Review articles, book chapters, and conference abstracts were also excluded to ensure that only high-quality primary sources were considered. 

The inclusion criterion was conducting research on leukemia cells. The studies collected utilized cell lines, animal models, or material derived from patients diagnosed with leukemia. Included studies were required to assess the therapeutic effect of exosomal miR in such material. Studies conducted on non-leukemic material or those evaluating the diagnostic significance of miR were not eligible for inclusion. Additionally, only articles published within the past 10 years qualified for the study.

The collected publications were highly diverse. For this reason, the analysis was conducted in four subgroups. The groups were organized based on the type of leukemia. However, due to the limited number of publications, it is not possible to compare studies within a single type of miR or the same type of material. Therefore, to increase the certainty of the evidence, further research is necessary.

### 2.4. Data Collection and Selection Process

Source selection was conducted in two phases. In the first phase, duplicates were removed using free online software, followed by manual double-checking. After this, titles, abstracts, and keywords were screened to identify those addressing the two main concepts and to exclude non-English sources. In the second phase, the abstracts of the remaining sources were reviewed to assess their relevance to the study’s topic and to determine whether microRNA analysis was included. This screening process enabled the reviewers to select articles that aligned with the study’s aims and inclusion criteria. 

The selection of articles was conducted independently by two reviewers and then cross-checked by two additional reviewers. No inconsistencies emerged between reviewers during the selection process. 

### 2.5. Data Items and Effect Measures

Data collected from the selected articles included information on the type of material used in the study, specifying whether it originated from humans, animals, or cell lines, along with the specific cell line type or animal model. The type of leukemia was also categorized into CLL, CML, ALL, and AML. Additionally, the type of miR evaluated was noted, along with its target. The impact of miR on the patient or cell was also considered, assessing characteristics such as its effect on cell proliferation and migration, as well as interactions with factors like tumor aggressiveness or chemotherapy response.

Due to the broad assessment of miR impact on leukemia, effect measures were evaluated using various techniques. These included levels of prognostic markers, inflammatory cytokine levels, counts of apoptotic cells, and cell motility. 

### 2.6. Study Risk of Bias Assessment

The Mixed Methods Appraisal Tool (MMAT) was used to assess the risk of bias. In this process, the collected studies were categorized as qualitative, quantitative non-randomized, quantitative descriptive, or mixed methods. Within each study type, questions assessing the quality of the studies were answered. A “yes” response was awarded 1 point, while a “no” or “can’t tell” response received 0 points. The risk of bias assessment was conducted by two reviewers.

## 3. Results

### 3.1. Study Selection

**Figure 2:**
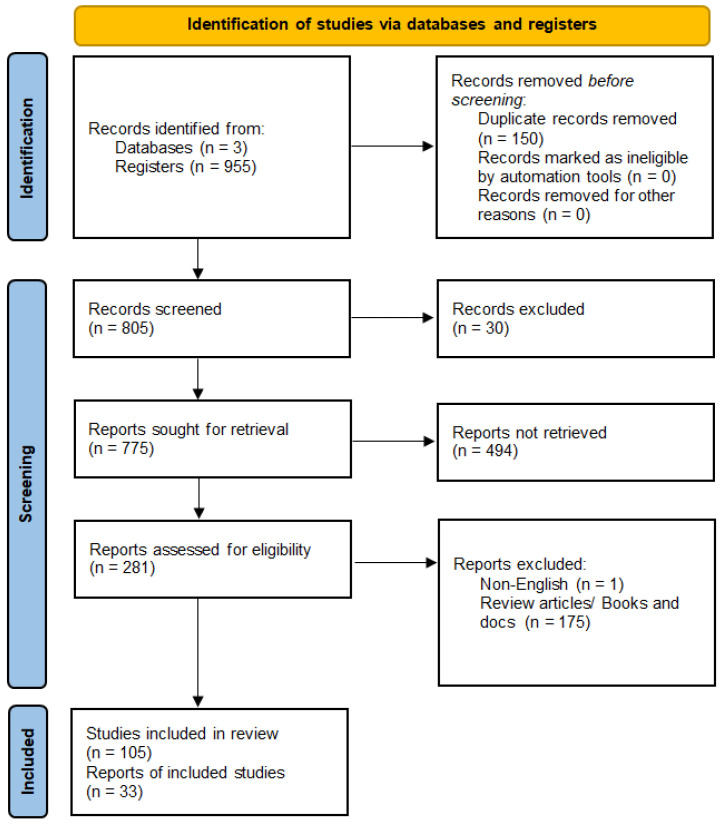
PRISMA 2020 flow diagram for new systematic reviews which included searches of databases and registers only [26].

The initial database search yielded 955 results, which were filtered in the first phase by removing duplicates and unrelated topics. In the next phase, 805 sources underwent full-text analysis for relevance and methodological rigor, leading to the exclusion of 700 sources. In the next step, review articles and genetic profile studies were eliminated, and articles describing the possible impact of miRNA on the pathomechanism of various types of leukemia were selected, resulting in a total of 33 articles included in the scoping review (Figure 2).

This review focuses on the potential therapeutic application of microRNA, rather than its role in the diagnostic process. Additionally, studies that were not conducted on leukemia cells were rejected. From the remaining publications, for studies conducted on cell lines, those were selected in which leukemia cells were cultured under similar conditions to ensure the most homogeneous research material possible. For this reason, publications based on the cultivation of leukemia cells under hypoxic conditions were excluded.

The majority of the studies only included AML, nine were about ALL, seven articles mentioned CML, and five regarded CLL.

### 3.2. Study Characteristics

**Figure 3:**
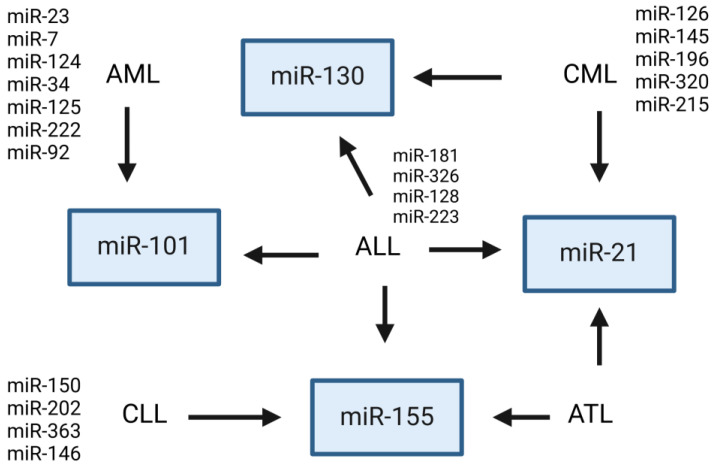
Most frequent miRNAs showing dysregulated expression in myeloid or lymphoid leukemia (created in BioRender.com (accessed on 3 November 2024), agreement number: JH27I62WZR)) https://BioRender.com/v60a038 accessed on 3 November 2024.

A total of 33 publications meeting the established criteria were collected. The studies utilized material from both patients and cell lines as well as animal models. The publications covered four types of leukemia: AML, ALL, CML, and CLL. The research focused on the significance of miRNA carried by exosomes in the pathomechanism of leukemia and its potential use in therapy (Table 1, Figure 3).

### 3.3. Risk of Bias in Studies

The MMAT [27] was employed to evaluate the quality of each study in this systematic literature review, allowing for a thorough assessment of both qualitative and quantitative research. The quality ratings of the included studies, as determined by the MMAT, are presented in Table 2.

**Table 1:** Characterization of the collected studies by comparing the publications in terms of the type of leukemia assessed, the material used, the type of miRNA, and its properties.

Author, Date, Reference	Material	Type of RNA Evaluated	miRNA Target	Impact of the Evaluated RNA on the Patient/Cells
CLL				
Stamatopoulos, 2015 [28]	Plasma and B lymphocytes of patients	miR-150	–	miR-150 level is associated with tumor aggressiveness
Yeh, 2015 [29]	Patients’ B lymphocytes	miR-150	–	miR-150 level is associated with tumor aggressiveness [23]
		miR-155	–	Influence on disease aggression and weakening of response to chemotherapy
Farahani, 2015 [30]	B lymphocytes from patients, MEC1 and HS5 cell lines	miR-202-3p	Sufu	Suppressive effect on tumor development
Smallwood 2016 [31]	Peripheral blood of patients, CD19+ mononuclear cells	miR-363	CD69	Critical role in the regulation of T-cell motility and immune synapse signaling function
Paggetti 2015 [32]	Bone marrow stem cells from patients	miR-146a, miR-155	–	Increased stromal cell proliferation, migration, and inflammatory cytokine secretion
CML				
Taverna, 2014 [33]	LAMA84 cell lines	miR-126	CXCL12 i VCAM1	Reduction in LAMA84 cell migration and adhesion
Chen, 2022 [34]	Bone marrow from patients, K562 cell lines, NOD-SCID mice	miR-145a-5p	USP6	Increases imatinib-induced K562 apoptosis
Taverna, 2015 [35]	K562 and LAMA84 cell lines, SCID mice	miR-21	PTEN	Increases VEGF secretion, formation of larger cell colonies
		miR-196b	–	Decreased Bcr-Abl protein level
Gao, 2019 [36]	Bone marrow and peripheral blood of patients, K562 and LAMA84 cell lines, SCL-tTa X TER-BCR/ABL mice	miR-320	BCR/ABL	Inhibition of K562 proliferation
Chai, 2023 [37]	Bone marrow and peripheral blood of patients, cell lines HEK293T, HL60, K562, BALL-1, and Jurkat cells	miR-130a/b	Cx43	Increases the immunosuppressive properties of cells, supports immune escape in tumors
Ohyashiki 2016 [38]	Peripheral blood from patients	miR-215	–	Imatinib therapy causes miR-215 expression levels to decrease, maintaining undetectable minimal residual disease (UMRD)
Taverna 2016 [39]	HUVEC cell lines	miR-21	PTEN	Decreased expression of anti-apoptotic Bcl-2 and decreased expression of WT1, growth of leukemic cells by decreased expression of PTEN
		miR-15a i miR-16	Bcl-2, WT1	
ALL				
Huang, 2022 [40]	Mouse cell lines L1210 and p388 and DBA/2 mice	shRNA—RNA synthetic	PD-L1	Increasing immunological properties, increasing the lifespan of mice
Yan, 2021 [41]	Patients’ peripheral blood, BALL-1 cell line	miR-181b-5p	–	Increased proliferation and migration and decreased apoptosis of ALL cells
Chai, 2023 [37]	Bone marrow and peripheral blood of patients, cell lines HEK293T, HL60, K562, BALL-1 and Jurkat cells	miR-130a/b	Cx43	Increases the immunosuppressive properties of cells, supports immune escape in tumors
Saffari 2024 [42]	Peripheral blood of patients, CD10 − /CD34 − cell lines, RN95, Nalm6 cell lines	miR-326	–	Cancer cell viability was dramatically suppressed in an exosomal miRNA dose-dependent manner
Rzepiel 2023 [43]	Platelet-free plasma (PFP)	miR-128-3p	–	Reduced expression positively correlates with minimal residual disease (MRD) in bone marrow flow cytometry at day 15 of treatment (potential therapeutic marker)
Habiel 2021 [44]	SCID/Bg mice	miR-101-3p, miR-193b-3p, miR-21-5p, miR-34a-5p	MMR, BRCA1	Marked reduction in the expression of components of the mismatch repair (MMR) pathway and BRCA1 (divergence of leukemic cells located in their microenvironment and the generation of therapy resistance)
Haque 2020 [45]	Cell lines M1, SUP-B15, NALM-6, REH, NALM-16	miR-181	–	Role in chemoresistance in relapsed leukemia
Colangelo 2022 [46]	CUTLL1 cell lines	miR-223-3p	NOTCH1	Increased population of resistant T-lymphocytic leukemia cells in response to conventional therapies [47]
AML				
Cheng, 2021 [48]	Blood from patients, cell lines Kasumi-1, HL-60, THP-1, HMSC, and bone marrow cells	miR-23b-5p	TRIM14	Increased apoptosis of THP-1 cells
Jiang, 2022 [49]	Patient plasma, cell lines HL60, THP1, U937, KG-1, MOLM13, MV4-11, GM12878, B-NDG mice	miR-7-5p	OSBPL11	Limiting proliferation and stimulating cell apoptosis
Otmani, 2023 [50]	Peripheral blood from patients	miR-24-3p	DENN/MADD	Increased T-cell apoptosis
Zhao, 2019 [51]	Cord blood from healthy women, cell lines HL-60, Molm-14, OCI-AML3, ML-2	miR-4532	LDOC1	Inhibition of hematopoiesis
Xu, 2020 [52]	KG-1a cell line	hsa-miR-124-5p	SMC4	Decreased proliferation and inhibition of the KG-1a cell cycle and increased apoptosis of KG-1a
Taniguchi, 2022 [53]	HL-60 and HL-60/ADR cell lines	miR-484	–	Increased cell proliferation
Hu, 2020 [24]	Bone marrow from patients, cell lines THP-1, KG1a, KASUMI-1	miR-34a	DHAC2	Reduction in cell proliferation, increase in apoptosis of leukemia cells, prolongs survival time [54]
Jiang 2018 [55]	Peripheral blood from patients	miR-125b	–	Higher risks of relapse and overall death
Yoshida 2019 [56]	Bone marrow stem cells, HTS-5 cells	miR-7977	signaling pathway Hippo-YAP	Inhibits the Hippo-YAP signaling pathway in bone marrow stem cells, spreading functionally impaired MSCs
Li 2022 [57]	Peripheral blood of patients	miR92a	PTEN, signaling pathway Wnt/β-catenin	Reduction in PTEN expression promotes cytarabine resistance in cells by activating the Wnt/β-catenin pathway
Yuan 2023 [58]	Bone marrow from patients	miRNA-222-3p	IRF2/INPP4B	Increased Th1/Th2 ratio and promotes apoptosis
Barrera-Ramirez 2017 [59]	Bone marrow from patients	miR-26a-5p i miR-101-3p	GSK3β, EZH2	Phosphorylation of GSK-3β in AML may activate the Akt pathway and is associated with poorer overall survival; genomic loss of EZH2 may lead to epigenetic changes and overexpression of HOX genes
		miR-23b-5p, miR-339-3p i miR-425-5p	APOBEC3A	-
Li 2022 [60]	U937 cell line	miR-3064-3p, miR-339-5p	p62	Increased expression of p62 may promote the maturation of AML cells into granulocytes, depending on NF-κB activation, predicting poor prognosis in AML
ATL (adult T-cell leukemia/lymphoma)				
El-Saghir 2016 [61]	Peripheral blood of deceased patients (frozen mononuclear cells), leukemia cell lines (Molt-4, C81, and HuT-102)	miR-21, miR-155	signaling pathway NF-κB	Changes in cellular morphology, increased proliferation, and induction of gene expression of migration and angiogenic markers

**Table 2:** Assessment of the quality of retrieved publications using the Mixed Methods Appraisal Tool [27].

Studies	Criteria from the Mixed Methods Appraisal Tool																								
	1.1	1.2	1.3	1.4	1.5	2.1	2.2	2.3	2.4	2.5	3.1	3.2	3.3	3.4	3.5	4.1	4.2	4.3	4.4	4.5	5.1	5.2	5.3	5.4	5.5
Type of Study	Qualitative					Quantitative Randomized Controlled Trials					Quantitative Non Randomized					Quantitative Descriptive					Mixed Methods				
Taverna, 2014 [33]	1	1	1	1	1						0	1	1	0	1						1	1	1	1	1
Yan, 2021 [41]	1	1	1	1	1						1	1	1	0	1						1	1	1	1	1
Jiang, 2022 [49]	1	1	1	1	1						1	1	1	0	1						1	1	1	1	1
Taverna, 2015 [35]	1	1	1	1	1						0	1	1	0	1						1	1	1	1	1
Zhao, 2019 [51]	1	1	1	1	1						0	1	1	0	1						1	1	1	1	1
Farahani, 2015 [30]	1	1	1	1	1						0	1	1	1	1						1	1	1	1	1
Gao, 2019 [36]	1	1	1	1	1						1	1	1	1	1						1	1	1	1	1
Chai, 2023 [37]	1	1	1	1	1						0	1	1	0	1						1	1	1	1	1
Huang, 2022 [40]											1	1	1	0	1										
Stamatopoulos, 2015 [28]											1	1	1	0	1										
Yeh, 2015 [29]											1	1	1	0	1										
Cheng, 2021 [48]											1	1	1	0	1										
El-Saghir, 2016 [61]											1	1	1	0	1										
Otmani, 2023 [50]											1	1	1	0	1										
Jiang, 2018 [55]											1	1	0	1	1										
Ohyashiki, 2016 [38]											1	1	1	0	1										
Chen, 2022 [34]											1	1	1	0	1										
Xu, 2020 [52]											0	1	1	0	1										
Taniguchi, 2022 [53]											0	1	1	0	1										
Hu, 2020 [24]											1	1	1	0	1										
Yuan, 2023 [58]											1	1	1	0	1										
Taverna, 2016 [39]											1	1	1	1	1										
Colangelo, 2022 [46]											1	1	1	1	1										
Saffari, 2024 [42]											1	1	1	1	1	1	1	1	0	1					
Barrera-Ramirez, 2017 [59]											1	1	1	0	1	1	1	1	0	1					
Smallwood, 2016 [31]											1	1	1	0	1	1	1	1	0	1					
Habiel, 2016 [44]											1	1	1	1	1						1	1	0	0	1
Yoshida 2019 [56]																1	0	1	0	1					
Rzepiel, 2023 [43]																1	0	1	0	1					
Li, 2022 [20]																1	1	1	0	1					
Li, 2022 [57]																1	1	1	0	1					
Haque, 2020 [45]																1	1	1	0	1					
Paggetti, 2015 [32]																1	0	1	0	1					

1. Qualitative: 1.1. Is the qualitative approach appropriate to answer the research question? 1.2. Are the qualitative data collection methods adequate to address the research question? 1.3. Are the findings adequately derived from the data? 1.4. Is the interpretation of results sufficiently substantiated by data? 1.5. Is there coherence between qualitative data sources, collection, analysis, and interpretation? 2. Quantitative randomized controlled trials: 2.1. Is randomization appropriately performed? 2.2. Are the groups comparable at baseline? 2.3. Are there complete outcome data? 2.4. Are outcome assessors blinded to the intervention provided? 2.5 Did the participants adhere to the assigned intervention? 3. Quantitative non-randomized: 3.1. Are the participants representative of the target population? 3.2. Are measurements appropriate regarding both the outcome and intervention (or exposure)? 3.3. Are there complete outcome data? 3.4. Are the confounders accounted for in the design and analysis? 3.5. During the study period, is the intervention administered (or exposure occurred) as intended? 4. Quantitative descriptive: 4.1. Is the sampling strategy relevant to address the research question? 4.2. Is the sample representative of the target population? 4.3. Are the measurements appropriate? 4.4. Is the risk of nonresponse bias low? 4.5. Is the statistical analysis appropriate to answer the research question? 5. Mixed methods: 5.1. Is there an adequate rationale for using a mixed methods design to address the research question? 5.2. Are the different components of the study effectively integrated to answer the research question? 5.3. Are the outputs of the integration of qualitative and quantitative components adequately interpreted? 5.4. Are divergences and inconsistencies between quantitative and qualitative results adequately addressed? 5.5. Do the different components of the study adhere to the quality criteria of each tradition of the methods involved? [27].

## 4. Discussion

In recent years, there has been increasing interest in non-coding RNAs of exosomal origin in cancers [18,62,63] due to their stability and non-invasiveness and also, as it seems, high value [18,63,64]. Many studies have presented evidence for the value of exosomal non-coding RNAs as diagnostic and prognostic/predictive biomarkers in leukemias. Currently, the role of exosomal non-coding RNAs as biomarkers focuses mainly on their value as diagnostic and prognostic factors, but more and more studies show that exosomes derived from cancer cells participate in immune suppression, angiogenesis, and metastasis [18,63,65,66,67,68,69].

### 4.1. Therapeutical Potential of Exosomal miRNA in Leukemia

In this review, we want to show that therapeutic implications are also possible. The therapeutic application of exosome miRNAs can be considered in many ways. Available studies indicate the possibility of using exosome miRNAs and their expression profile in assessing the response to therapy, but they can also have therapeutic applications, for example in immunotherapy, combination therapies, and as drug delivery systems [66,70,71]. Specific miRNAs of exosomal origin can be therapeutic targets by targeting their function inhibition, potentially leading to reduced leukemia cell proliferation and increased apoptosis. Also, targeting specific ncRNAs or their pathways can increase the sensitivity of leukemia cells to existing treatments. Another important aspect is the potential to change the tumor microenvironment, which will allow for sensitizing immune cells to the developing tumor [70,71]. In recent years, evidence has emerged that communication between different cell types of the immune microenvironment and tumor cells is mediated by exosomes [72,73]. Increasing evidence indicates that the tumor microenvironment is not only a result of cancer development but rather contributes to it [69,71]. Exosomal miRNAs can affect the tumor microenvironment by modulating immune responses and promoting tumor growth or metastasis and therefore represent targets for cancer immunotherapy [31,32,50,61,73]. The incubation of leukemic exosomes, derived from cell culture supernatants or patient plasma, with human stromal cells shows that they are readily taken up into endosomes and induce gene expression [30]. These findings underscore the contribution of leukemia-derived exosomes to cellular transformation and their potential value as biomarkers and targets in therapeutic strategies. The development of therapeutic strategies based on blocking defined miRNAs or protecting their targets is a rational approach worth considering. In the works on the use of exosomes, special attention is paid to minimizing systemic toxicity while maximizing therapeutic efficacy and enhancing anti-tumor immune responses [74,75]. Many works indicate the effectiveness of exosomes as a therapeutic agent in cancer therapy [76].

### 4.2. Exosomal miRNA in Leukemia: Pathogenesis and Therapy

#### 4.2.1. Acute Myeloid Leukemia

Among non-coding RNAs of exosomal origin, the majority of research focuses on miRNAs. Consequently, this review emphasizes the therapeutic implications of exosomal miRNAs in leukemias, particularly in AML. Several exosomal miRNAs, such as miR-7-5p, miR-23b-5p, miR-24-3p, miR-26a-5p, miR-34a, miR-92a, miR-101-3p, miR-124-5p, miR-125b, miR-222-3p, miR-339-3p, miR-339-5p, miR-425-5p, miR-3064-3p, miR-4532, and miR-7977, have been identified as potential therapeutic targets in AML [48,49,50,51,52,53,54,55,56,57,58,59].

Research by Yoshida et al. highlights exosomal miR-7977 as a key regulator of the Hippo-YAP signaling pathway in BM-MSCs, which may influence the leukemia-supporting stroma and serve as a therapeutic target in AML [56]. Otmani et al. demonstrated that exosomal miR-24-3p plays a role in regulating cell proliferation and apoptosis, with its overexpression linked to poor prognosis and disease relapse in AML. This miRNA contributes to cancer tolerance by affecting T lymphocytes in AML patients, suggesting that blocking exosomal miR-24-3p could lead to innovative immunotherapy strategies [50].

Li et al. identified exosomal miR-92a in plasma as a contributor to resistance to Ara-C in MDS and AML, suggesting it as a potential therapeutic target. Their studies also revealed that miR-3064-3p and miR-339-5p, derived from p62-knockdown AML cells, exhibited reduced expression, further indicating that exosomal miRNAs could be a target for therapy. Additionally, Li’s team emphasized that AML-derived exosomes promote angiogenesis [57].

Barrera-Ramirez et al. highlighted the significance of exosomal miR-26a-5p, miR-101-3p, miR-23b-5p, miR-339-3p, and miR-425-5p in leukemogenesis, disease progression, and as potential therapeutic targets in AML [59]. Hu et al. reported that targeting increased miR-34a expression to induce leukemia stem cell death could be an effective AML treatment strategy [24,54]. Similarly, Jiang et al. suggested that exosomal miR-7-5p, derived from BMSCs, inhibits AML proliferation and promotes apoptosis, supporting the use of BMSC-derived exosome-based therapies for AML [49].

Chen et al. postulated that exosomal miR-23b-5p from human mesenchymal stem cells could inhibit cancer cells and serve as a therapeutic target for AML [48]. Yuan et al. found that exosomal miR-222-3p promotes AML cell apoptosis by regulating IRF2 expression, identifying it as another potential therapeutic target [58]. Xu et al. demonstrated that bone marrow-derived exosomes, through miR-124-5p, promote apoptosis and inhibit cell proliferation and cycle progression, suggesting their potential in AML treatment [52].

Zhao et al. showed that exosomal miR-4532, derived from AML cells, inhibits normal HSC hematopoiesis, further highlighting its therapeutic relevance [51]. Lastly, Jiang et al. demonstrated that exosomal miR-125b concentration could serve as an independent prognostic indicator for AML, with elevated levels linked to higher relapse rates and mortality [55].

#### 4.2.2. Chronic Lymphocytic Leukemia

When assessing the work on CLL, it can be seen that the main therapeutic targets indicated by the researchers are the following exosomal microRNAs: miR-21, miR-29, miR-145a-5p, miR146a, miR-150, miR-155 miR-202-3p, miR-223, and miR-363 [28,29,30,31,32,34,61]. 

Pagetti et al. showed that CLL-derived exosomes (CLL-EEVs) actively promote disease progression by modulating several functions of the surrounding stromal cells, which acquire the characteristics of cancer-associated fibroblasts, thus creating a niche promoting the adhesion, survival, and growth of CLL cells. The researchers noted that the most abundantly expressed miRNAs in exosomes were, among others, miR-21, miR-155, and miR146a [32]. Also, El-Saghir et al. in their studies point to miR-21 and miR-155 of exosomal origin as those that change cell properties to create a more favorable environment for leukemia. The authors also emphasize the important role of exosomal-derived vascular endothelial growth factor in leukemogenesis [61]. The study by Yeh et al. identified exosomal miR-29, miR-150, miR-155, and miR-223 as important for the profile and therapy of CLL [29]. Also, in the work by Stamatopolous, miR-150 of exosomal origin is indicated as an important potential target of therapy in CLL [28]. Smallwood et al., in their studies, showed that CLL-EVs have the ability to modify T-cell function by increasing the speed of motility, signaling of immune synapses, and interactions with tumor cells. The authors show that the treatment of autologous CD4 + CD40 / IL-4 T lymphocytes with CLL-EVs derived from miR-363 knockdown cells blocked the modulation of T-cell migration speed [31]. Chen et al. showed that the administration of exosomes to human umbilical cord MSCs promoted imatinib-induced cell apoptosis via miR-145a-5p, seeing in this exosomal miR new therapeutic strategies in drug-resistant leukemia [34]. Exosomal miR-202-3p has been identified as an important player in CLL biology and a potential therapeutic target, causing increased expression of the Hedgehog (Hh) signal in CLL stem cells [30].

#### 4.2.3. Acute Lymphoblastic Leukemia

As potential therapeutic targets for ALL, the authors indicate exosomal miRNAs: miR-17-92a, miR-128-3p, miR-181a, miRNA-181b-5p, and miR-326 [41,42,43,45,46,55]. The role of the selected exosomal miRNAs in monitoring the efficacy of therapy or assessing residual disease in leukemias is also of great importance. Rzepiel et al. suggest that miR-128-3p in the exosome-enriched fraction may be a valuable biomarker for tracking bone marrow function or response to therapy in ALL [43]. Colangelo et al. showed that exosomes secreted by T-ALL contain NOTCH1-dependent miRNAs (mainly miR-17-92a). They control pathways promoting the expansion and survival of highly proliferative human T-cell leukemia cells and also regulated genes that characterize patients with relapsed and refractory early T-cell progenitors of ALL. Studies conducted by Colangelo demonstrated the ability of exosomal miR-17-92 to propagate molecular information among T-ALL cells, which was able to restore, at least partially, the defective NOTCH1 signaling pathway [46]. Increasingly, authors see exosomal miRNAs as a source of drug resistance in leukemias. Haque et al., in their studies, show that the exosomal non-suppressive nature of miR-181a ALL in relapsed leukemia may contribute to resistance to chemotherapy. Researchers suggest a potential role of miR-181a inhibitor along with chemotherapy in the treatment of relapsed leukemia [45]. Habiel et al. suggest that targeting MMR and BRCA1 by exosomal miRNAs may help stabilize the genome of leukemic cells and subsequently reduce resistance to treatment [44]. In turn, Saffari et al. show that the assessment of exosomal miR-326 may be a new way to diagnose primary resistance to treatment in childhood ALL and also a potential therapeutic target for ALL [42]. Exosomal miRNA-181b-5p has also been indicated as a malignant factor in ALL cells and a potential therapeutic target [41].

#### 4.2.4. Chronic Myeloid Leukemia

Among potential therapeutic targets of CML, mainly exosomal miR-21, miR-126, miR-215, miR-32, and miR-484 have been indicated [33,35,36,37,38,39,53]. Taverna et al. show that curcumin treatment changes the molecular properties of CML exosomes, induces the release of exosomes enriched in antiangiogenic proteins and depleted in proangiogenic proteins, and modulates the organization of the endothelial barrier. Curcumin-stimulated CML exosomes attenuated the effect of CML exosomes on the endothelium due to changes in their proteomic composition and in the transport of miR-21 [39]. The selective packaging of miR-21 in exosomes may contribute to the antileukemic effect of curcumin in CML [35]. The authors also indicate the potential use of exosome-derived miR-126 in CML therapy [33]. In turn, Ohyashiki et al. show that the assessment of exosomal miR-215 may play a role in monitoring the effective discontinuation of imatinib in patients with CML [38]. Taniguchi et al. show that the increase in exosomal miR-484 is associated with the acquisition of drug resistance and the increased proliferation of leukemic cells [53]. In turn, studies conducted by Gao indicate that exosomal miR-320 is also an important mediator of leukemia progression and is a potential therapeutic target for CML [36].

### 4.3. Most Frequent Exosomal miRNAs Showing Dysregulated Expression in Leukemia

The main issues raised by researchers in the context of the possibility of using exosomal ncRNAs in the therapy of leukemia include the influence of miRNAs on the modification of the microenvironment of leukemic cells through the ability to modify T lymphocytes in patients with leukemia [31,32], the influence on angiogenesis processes [39,60], and the influence on important signaling pathways from the point of view of leukemogenesis, such as NF-κB, Wnt/β-catenin, and NOTCH [46,57,61]. Another important aspect indicated is the possibility of using exosomal miRNAs to monitor treatment progress [43]; using co-therapy strategies to overcome drug resistance [29,34,38,45]; the immunotherapy of leukemias [31,46]; influencing cell proliferation, migration, adhesion, and apoptosis [24,32,33,36,41,49,53,54,58,61]; and the potential use of exosomal ncRNA vaccines [40]. The exosomal miRNAs that appeared most frequently as potential therapeutic targets in different types of leukemias were miR-21 [35,39,44,61], miR-23b-5p [48,59], miR-34 [24,44,54], miR101-3p [44,59], miR-150 [28,29], and miR-155 [26,46,55]. It seems that the indicated exosomal miRNAs have the greatest therapeutic potential. The results obtained by the researchers were consistent. Both Habiel and Hu assessed the role of miR-34 in leukemia. Although the studies concerned other types of leukemia, their results unanimously confirm that higher expression of miR-34 may have a therapeutic effect [24,44,54]. Four groups of researchers investigated the effect of miR-21 on the course of CML and ALL. Habiel shows that targeting miR-21 can reduce resistance to therapy [44]. El-Saghir observed that miR-21 increases proliferation, migration, and angiogenesis, which are factors favoring tumor development [61]. Taverna reached similar conclusions, showing the anti-apoptotic and proangiogenic effects of miR-21 [35,39], indicating that exosomal miR-21 is a potential candidate for use in new therapeutic strategies. Additionally, Stamatopoulos showed that miR-150 affects tumor aggressiveness [28]. This is also confirmed by Yeh’s studies [29]. Moreover, miR-155 has been evaluated in three studies. All of them unanimously confirm its negative effects on leukemias. Yeh showed that miR-155 increases tumor aggressiveness and decreases response to treatment [29], El-Saghir observed increased proliferation, migration, and angiogenesis [61], and Paggetti showed increased secretion of proinflammatory cytokines [32]. In the case of studies evaluating the function of miR-101-3p, Barrera-Ramirez showed that miR-101-3p increases disease progression [59]. Habiel, on the other hand, showed that targeting miR-101-3p can reduce resistance to treatment [44]. It should be noted that different groups of researchers evaluated different types of leukemia.

This review focuses mainly on the role of exosomal miRNA with the potential for use in leukemia therapy. However, other ncRNAs, including circRNA and lncRNA, also show therapeutic potential in leukemia. lncRNAs are desirable therapeutic targets in cancer treatment [77,78]; however, in the context of leukemia therapy, this information is quite scarce. The study by Xiao et al. indicates that circulating exosomal LINC00265, LINC00467, UCA1, and SNHG1 may act as promising cell-free biomarkers for monitoring AML treatment [79]. The potential for circRNA use is briefly characterized in the next subunit.

### 4.4. Circular RNA in Leukemia: Pathogenesis and Therapy

There are ncRNAs that have been confirmed as key molecules participating in the pathogenesis of leukemia. Their abnormal expression might be used to classify leukemia subtypes, evaluate the prognosis, and predict the response to chemotherapy. circRNAs are a group of recently discovered ncRNAs associated with many diseases. circRNAs are a class of ncRNAs characterized by their covalently closed loop structure. Unlike linear RNAs, circRNAs do not have 5′ caps or 3′ poly-A tails, which makes them more resistant to exonucleases and potentially more stable [80]. Their roles in gene regulation and cellular processes have garnered significant interest, particularly in cancer research [81,82,83].

The role of circRNAs in the pathogenesis of leukemias seems to be quite broad. circRNAs can regulate gene expression, modulate signaling pathways, impact leukemia subtypes, and function as biomarkers.

CircRNAs can function as sponges for miRNAs, thereby influencing the availability of miRNAs to their target mRNAs [84,85]. This mechanism can affect the expression of genes involved in leukemia pathogenesis. For instance, circRNAs that act as miRNA sponges can modulate the activity of oncogenes or tumor suppressor genes, contributing to leukemogenesis [84,85,86,87].

Certain circRNAs are involved in key signaling pathways associated with leukemia. They can regulate pathways such as the Wnt/β-catenin, Notch, and NF-κB pathways, which are crucial in maintaining hematopoietic stem cell homeostasis and promoting malignant transformation [81,88,89,90].

Research has identified specific circRNAs associated with various subtypes of leukemia, including AML and CLL. These circRNAs often exhibit differential expression patterns that correlate with disease progression, prognosis, and response to treatment [84,85].

Due to their stability and distinct expression profiles, circRNAs hold potential as biomarkers for leukemia diagnosis and prognosis. Their presence in body fluids like plasma and serum makes them promising candidates for non-invasive diagnostic and prognostic tools [80,84,85,86,91,92].

Targeting circRNAs offers a novel therapeutic strategy in leukemia. Strategies include designing molecules that specifically degrade or modulate the function of oncogenic circRNAs or restore the function of tumor-suppressive circRNAs. siRNAs and antisense oligonucleotides are potential tools for these purposes [80,84,85,86]. The development of advanced drug delivery systems can enhance the targeted delivery of circRNA-based therapeutics. Nanoparticles and liposomes can be engineered to deliver circRNA modulators specifically to leukemia cells, thereby increasing treatment efficacy and minimizing off-target effects.

Combining circRNA-based therapies with existing treatments, such as chemotherapy or targeted therapies, could improve therapeutic outcomes. For instance, circRNA modulators could sensitize leukemia cells to conventional drugs or overcome resistance mechanisms. It was shown that the combination of circKAT6A, circLNPEP, circMDM2, and circMYH9 could successfully discern CLL cells from healthy B-lymphocytes and differentiate patients with early CLL from those in advanced stages [81].

Ongoing research and clinical trials are exploring the role of circRNAs in leukemia therapy. Advances in understanding circRNA biology and its functional implications in leukemia are expected to pave the way for innovative treatment strategies and personalized medicine approaches. With the discovery and identification of more circRNAs, as well as the confirmation of more functional experiments and clinical trials, the emergence of circRNA-related targeting therapy of leukemia will be significantly accelerated, realizing the successful conversion of basic medicine to clinical practice [80]. It was found that e.i. circNPM1 could promote the resistance of AML cells to adriamycin via the miR-345-5p/FZD5 axis, indicating that circNPM1 was a potential marker in AML drug resistance therapy [84,85,93].

Circular RNAs may play a significant role in the pathogenesis and therapy of leukemia. Their involvement in gene regulation and signaling pathways and their potential as biomarkers and therapeutic targets highlight their importance in advancing leukemia research and treatment. Continued exploration of circRNA biology and innovative therapeutic approaches will be key to improving outcomes for leukemia patients.

### 4.5. Potential Therapeutic Applications of Exosomal-Derived miRNAs

The potential use of exosome-derived miRNA in a patient with leukemia is a complex process that involves many issues. First of all, appropriate technical preparation, ensuring patient safety, and ethical, regulatory, and procedural considerations should be considered. The involvement of many teams of researchers is crucial for the development of therapies based on exosome-derived miRNA [76,94].

The potential application procedure should include several important steps. First of all, attention should be paid to the method of obtaining exosomes from the patient [95,96]. There are many isolation methods that have different efficiencies and different applications in specific clinical cases, from the most commonly used ultracentrifugation [97], through commercially available isolation kits, to precipitation [98]. An important aspect here, apart from the method of obtaining exosomes, is the selection of the appropriate patient body fluid from which these exosomes are to be isolated [99,100]. In the next stage, the isolated exosomes must be properly characterized, which also poses many difficulties. Exosomes are quite heterogeneous in terms of size but also content and surface markers, and they also differ in terms of their collection source [14,101,102,103]. It is worth noting here that the isolation of exosomes and their purification from body fluids is technically difficult, which actually hinders the reproducibility of the procedure, which is so important in the clinical context [100]. A significant challenge currently facing researchers is the development of an improved high-throughput isolation technique and a faithful analysis of the exosome charge in order to increase their therapeutic utility [74]. The next important issue is the proper extraction of miRNA from exosomes. There are many commercially available kits on the market that offer the possibility of isolation with high miRNA recovery. Next, the appropriate modification of miRNA, i.e., knockdown or overexpression in target exosomes depending on clinical needs. Exosomes must be modified to transport miRNAs that have tumor-suppressing effects. Next, the extracted and modified miRNAs must be formulated for delivery. However, before such modified exosomes can be used, their functional validation is necessary. Before use, it should be confirmed that the isolated/modified miRNAs have the desired biological effects in vitro [96,104].

For the purpose of application, it is also necessary to determine the appropriate load and appropriate route of administration for the patient to obtain the appropriate action at the target site and the desired effect. Available studies show that exosomes can be administered by at least four different routes: intranasal, intravenous, intraperitoneal, and intracranial [99]. In addition to choosing the right method of administration, it is equally important to determine the stability of exosomes and how they are stored, perform sterility tests, obtain market authorization, verify compliance with regulations and appropriate quality, and confirm the efficacy and safety of use [105]. After potential use, further monitoring of the patient is necessary for therapeutic results and possible side effects [74,99,105,106].

### 4.6. Exosome Engineering

Exosome engineering focuses mainly on the therapeutic targeting of exosomes themselves by modifying their cargo and the exosomal enhancement of the body’s anti-cancer immunity [74]. Cell-free exosome therapy can consist of both the direct loading of exosomes with broadly understood therapeutic agents, enhancing existing treatment methods, and indirectly through genetic modification of exosomes collected from the patient or even artificially created therapeutic exosomes. The methods used to target ncRNA include antisense oligonucleotides or RNA interference technology [78]. It has been shown in animal models that exosomes can effectively deliver miRNA to cancer cells [107]. Due to their properties, exosomes can potentially avoid degradation in lysosomes or phagocytosis by macrophages and are therefore used to transport various molecules, including nucleic acid fragments [104]. There are various techniques for cargo loading, including preproduction loading methods such as electroporation, transfection, and co-incubation [105]. However, packaging cargo into exosomes in the desired amount remains a challenge to this day. Exosomes can be modified not only genetically but also biochemically to improve their therapeutic parameters and to selectively deliver exosomes to target cells for precise treatment [105,106]. An extremely important and recently raised topic is also the disruption of oncogenic signaling by inhibiting exosome biogenesis, secretion, and uptake [108]. The inhibition of exosome biogenesis and the secretion of cancer exosomes, or reducing their presence in the circulation, can be achieved by many different mechanisms, such as the use of exosome secretion inhibitors in combination with exosome therapy.

### 4.7. Clinical Trials

In clinical trials, exosomes in the therapeutic context are mainly evaluated for their ability to act as nanocarriers for drug delivery, but the number of these trials is small and currently includes cancers other than leukemia, including pancreatic cancer, colorectal cancer, and non-small-cell lung cancer (clinicaltrial.gov). Two studies are evaluating the potential use of exosomes as cancer vaccines. Currently, there are few studies in the context of leukemia therapy using exosomal therapy: NCT04460963, NCT03537599, and NCT06245746, which concerns the use of exosomes derived from umbilical cord MSCs to alleviate common chemotherapy-induced myelosuppression in patients with AML after achieving remission [109]. No clinical trials are currently evaluating the potential use of exosomal miRNAs in leukemia therapy. Research into the use of exosomes in clinical practice faces numerous challenges. The huge diversity of molecules, not fully identified mechanisms of their action, or many technical issues related to the preparation of exosomes and their administration to the patient definitely delay the possibility of their application. These include the need to standardize isolation and characterization methods, the reproducibility of preparations, and the demonstration of the safety and efficacy of these therapies [95]. Clinical results using exosomes are promising, reflecting research efforts aimed at maximizing their therapeutic potential in cancer treatment, but require rigorous compliance with good manufacturing practice [106,110,111,112], therefore most of the work related to the potential application of exosomal miRNA in cancer therapy is in the preclinical phase.

## 5. Conclusions and Future Directions

Non-coding microRNAs of exosomal origin and the possibility of their practical use have attracted great interest from scientists, especially in the context of cancer, including leukemia therapy. Analysis of non-coding RNAs in exosomes reflects the state of the stem cell, can support the diagnostic process, determine the prognosis, and be a therapeutic target. 

Before ncRNAs of exosomal origin can be used in therapy, their role requires a deeper understanding. Further studies are needed to explain how specific ncRNAs are involved in leukemogenesis, drug resistance, metastasis, and disease relapse and what the mechanisms of their action are. Further research in this field can lead to improved therapeutic strategies and better patient outcomes. In order to properly use the potential of ncRNAs in leukemia therapy, it is necessary to better understand their functions and consider the technical limitations of their use and regulatory and safety issues. Despite progress, the exact functional roles of many ncRNAs in leukemia are still not fully understood. Further studies are needed to explain the mechanisms of their action and interactions with other “molecular players”.

At present, it seems that therapeutic strategies targeting selected ncRNAs and the pathways in which they participate can increase the sensitivity of leukemia cells to existing treatment methods by directly influencing leukemia cells, such as reducing cell proliferation or inducing apoptosis and modifying the tumor microenvironment. 

Another challenge regarding the possibility of using exosomal ncRNAs is the immunotherapy of leukemias. Studies are being conducted on the use of exosomal ncRNAs in the potential to enhance the immune response against leukemia cells. 

Further studies should also focus on the possibility of using exosomes as potential drug delivery systems. Exosomes have a natural origin, quite high stability, and the ability to penetrate body barriers. They can be used as drug carriers directly to the tumor cell, which would increase the effectiveness of the therapies used and minimize side effects. 

A significant challenge in the therapeutic use of ncRNA from exosomes is standardization. Many studies are needed to standardize the methods of exosome isolation so that the studies conducted are comparable and reproducible in both scientific and clinical approaches. Techniques for detecting and quantifying ncRNAs need to be improved to improve sensitivity and specificity. Progress in RNA sequencing and bioinformatics is crucial for this purpose.

An important aspect before therapeutic application is to determine the efficacy of action, assess the safety profile including long-term safety, and indicate the necessary load/dose to achieve the desired therapeutic effect.

In summary, the use of EV-ncRNAs, especially miRNAs, in the therapy of leukemias is very promising. In the future, an approach based on exosomal ncRNAs may provide new innovative therapeutic strategies. However, the development of therapies based on exosomal ncRNAs requires careful consideration of safety and potential side effects. Comprehensive preclinical and clinical evaluations are necessary to ensure the safety and efficacy of these new therapies.
